# Severe Neurological Disorders in the Greenlandic Population: A Nationwide Register‐Based Study

**DOI:** 10.1002/brb3.71086

**Published:** 2025-12-22

**Authors:** Nete Munk Nielsen, Mikael Andersson, Melinda Magyari, Nils Koch‐Henriksen, Egon Stenager, Anders Koch

**Affiliations:** ^1^ Department of Epidemiology Research Statens Serum Institut Copenhagen Denmark; ^2^ Focused Research Unit in Neurology, Department of Neurology Hospital of Southern Jutland, University of Southern Denmark Aabenraa Denmark; ^3^ The Danish Multiple Sclerosis Registry, Department of Neurology Rigshospitalet Copenhagen Denmark; ^4^ Danish Multiple Sclerosis Center, Department of Neurology University of Copenhagen, Rigshospitalet Copenhagen Denmark; ^5^ Institute for Clinical Medicine University of Copenhagen Copenhagen Denmark; ^6^ Multiple Sclerosis Clinic of Southern Jutland (Aabenraa, Kolding, Esbjerg), Department of Neurology, Aabenraa Hospital of Southern Jutland, University of Southern Denmark Denmark; ^7^ Department of Infectious Disease Epidemiology and Prevention Statens Serum Institut Copenhagen Denmark; ^8^ Department of Infectious Diseases Rigshospitalet University Hospital Copenhagen Denmark; ^9^ Ilisimatusarfik University of Greenland Nuussuaq Greenland; ^10^ Department of Internal Medicine Queen Ingrid's Hospital Nuuk Greenland

## Abstract

**Background:**

Few studies have addressed the burden of neurological disorders in Greenland (GL). We aim to estimate nationwide incidences and prevalence of a broad group of neurological disorders in the total Greenlandic population and according to ethnicity (Inuit, Non‐Inuit). To explore the importance of ethnic and environmental factors we estimated corresponding rates among Inuit and Non‐Inuit living in Denmark (DK).

**Methods:**

A long‐standing collaboration between the Danish and Greenlandic healthcare system enabled us to follow the Greenlandic and Danish population for dementia, Parkinson's Disease (PD), epilepsy, stroke, and infections of the Central Nervous System using national registries from both countries. Incidence rates (IRs) were calculated using log linear Poisson‐regression for the combined period 1987‐2014, and stratified according to ethnicity, country of residence and periods. Age‐standardized IRs (ASIRs) were based on the WHO 2000–2025 standard population.

**Results:**

The Greenlandic IRs of epilepsy and ischemic stroke were 98.6 (95% CI: 93.8–104) and 118 (95% CI: 113–124) respectively, per 100,000 person‐years of risk. IRs for the remaining neurological disorders were below 40 per 100,000. During follow‐up we observed an increase in IRs of ischemic stroke and a less pronounced for dementia. Apart from dementia, ASIRs of neurological disorders were generally higher in the Greenlandic population compared with the Danish, most pronounced for subarachnoid hemorrhage (ASIR_GL_/ASIR_DK _= 2.36 (2.12–2.62)). Inuit in Greenland were at a lower risk of stroke, PD and especially dementia (ASIR_GL_/ASIR_DK _= 0.40 (0.35–0.46)) compared with Inuit in Denmark. The most prevalent neurological disorders in Greenland in 2014 were stroke and epilepsy.

**Conclusion:**

Our study emphasizes that stroke and epilepsy are important causes of morbidity in Greenland and suggests that dementia may become a challenge. Noticeable differences according to ethnicity and country of residence warrants further research.

## Graphical abstract

A long‐standing collaboration between the Danish and Greenlandic healthcare system including the transfer of patients in needs of specialized treatment to Danish hospitals, and the inclusion of all Greenlandic inhabitants in the Danish Civil Registration System enabled us to follow the total Greenlandic and Danish population for neurological diagnoses in Danish as well as Greenlandic nationwide health registers as far back as 1987 the year the Greenlandic National Patient Register was established (Figures are PowerPoint icons or created using IA)

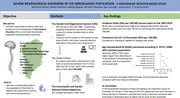

## 1 Introduction

Greenland, the world's largest island, has self‐government within the Danish Kingdom (Bundgaard et al. 2022; Statistics Greenland 2023). In the last 50–100 years, the Greenlandic society has been through a tremendous transition from a hunting society to a Nordic welfare state less dependent on hunting (Bjerregaard and Larsen 2018). Alongside this development, the Greenlandic population is getting older (Statistics Greenland 2023, Statbank Greenland 2017) and chronic and life‐style induced disorders are becoming an increasingly important cause of morbidity and mortality (Bjerregaard and Larsen 2018, Iburg et al. 2001). Accordingly, one would expect that also neurological disorders will become an increasing challenge for the future Greenlandic health care system (GBD 2015 Neurological Disorders Collaborator Group 2017).

Several studies have explored the burden of diseases in Greenland, but few have focused on neurological disorders. Thus, among a total of 640 health research publications concerning Greenland or Greenlanders from 2001 to 2020, only 13 studies concerned neurological disorders (Ipsen et al. 2024). Although, there are differences between the measures stroke has overall been found to be an important cause of morbidity and mortality in the Greenlandic population (Bjorn‐Mortensen et al. 2013; Kjaergaard and Bjerregaard 2004; Larsen et al. 2024; Svendsen et al. 2024; Bjerregaard et al. 2003; Bjorn‐Mortensen et al. 2013; Kjaergaard and Gelvan 2004; Kromann and Green 1980), an assumption which is in line with observations by clinicians and health‐care workers in Greenland (personal communication 2025). Less research has focused on the occurrence of other neurological disorders. However, the prevalence in Greenland of dementia, Parkinson's Disease (PD) and childhood epilepsy have been found to be lower (The Greenlandic Goverment 2012; Taudorf et al. 2019), almost 2–fold higher (Wermuth et al. 2004) and similar (Blichfeldt et al. 2004), respectively, to corresponding rates in Denmark, and the incidence rate of infections of the Central Nervous System (CNS) twice as high as that in Denmark (Nordholm et al. 2017).

Some of the above studies are, however, limited by few cases, selected study groups and periods, restricted geographical location and by not studying secular trends. Such information is useful for future prevention strategies and resource planning.

Recently, a collaboration group of neurological researchers, the Global Burden of Disease (GBD) studies developed a mathematical model with the purpose of estimating global as well as country‐specific rates, including Greenlandic, of morbidity and mortality of various neurological diseases (GBD Parkinson's Disease Collaborators 2018; GBD Dementia Collaborators 2019; GBD Multiple Sclerosis Collaborators 2019; GBD Epilepsy Collaborators 2019; GBD Meningitis Collaborators 2018; GBD Stroke Collaborators 2019). The model included a composite indicator of income per person, years of education, and fertility, the so‐called socio‐demographic index (SDI), in combination with data from previous studies and selected covariates depending on the neurological disorder studied (GBD Parkinson's Disease Collaborators 2018; GBD Dementia Collaborators 2019; GBD Multiple Sclerosis Collaborators 2019; GBD Epilepsy Collaborators 2019; GBD Meningitis Collaborators 2018; GBD Stroke Collaborators 2019).

Inspired by one of the above GBD studies predicting a surprisingly high prevalence of MS in 2016 in Greenland, we carried out a nationwide register‐based cohort study (Nielsen et al. 2024) and found a prevalence of MS in Greenland less than one‐fourth of that predicted by the GBD study (GBD Multiple Sclerosis Collaborators 2019). Theoretical disease measurements of prevalence or incidence from other of these GDB studies (GBD Parkinson's Disease Collaborators 2018; GBD Dementia Collaborators 2019; GBD Epilepsy Collaborators 2019; GBD Meningitis Collaborators 2018; GBD Stroke Collaborators 2019) addressing PD, dementia, epilepsy, stroke and meningitis are compatible with corresponding rates in some of previous Greenlandic studies (Bjorn‐Mortensen et al. 2013; Blichfeldt et al. 2004; Wermuth et al. 2004), but certainly not all (Larsen et al. 2024; Kromann and Green 1980; The Greenlandic Goverment 2012; Nordholm et al. 2017; Olesen 1996). As noted by the authors of the GBD studies data in many regions of the world, including Greenland, are sparse, accordingly some of the predicted rates should be interpreted with caution.

According to the beforementioned GBD studies the overall global burden of neurological disorders has increased substantially from 1990 to 2016, primarily due to population growth and aging (GBD 2015 Neurological Disorders Collaborator Group 2017; GBD 2016 Neurology Collaborators 2019). Thus, despite a decrease in mortality rates of stroke (GBD Stroke Collaborators 2019), meningitis (GBD Meningitis Collaborators 2018) and epilepsy (GBD Epilepsy Collaborators 2019), and a reduction in age‐standardized incidence rate of stroke (GBD Stroke Collaborators 2019) the absolute number of people affected by, dying or remaining disabled from neurological disorders has increased and neurological disorders is today considered as a major cause of death and disability worldwide (GBD 2015 Neurological Disorders Collaborator Group 2017; GBD 2016 Neurology Collaborators 2019).

The Greenlandic population is still younger than the European population (Galan 2025), but it has been predicted that 14% of the individuals living in 2040 will be 65 years of age or older, which is almost twice as many as in 2012 (Statistic Greenland 2012). The number of patients in Greenland who will need care by clinicians with expertise in neurological conditions will probably grow in the coming decades (GBD 2015 Neurological Disorders Collaborator Group 2017), placing an increased demand on the already overstretched resources of the Greenlandic health care system. For resource planning and possibly establishment of prevention strategies, nationwide realistic measures–for example, prevalence and incidence of the different neurological disorders in Greenland–are important.

To sum up, our understanding of the burden of neurological disorders in the Greenlandic population is still incomplete. The aim of the present study is therefore to obtain nationwide data on the incidence and prevalence of the most common neurological disorders (dementia, PD, epilepsy, stroke, and CNS infections) in the Greenlandic Inuit and Non‐Inuit population during a follow‐up period of almost 3 decades using register data. By comparing these estimates with the corresponding estimates in the Danish Inuit and Non‐Inuit population, we hope to get a better comprehension of the importance of genetic and environmental factors in the etiology of the above neurological disorders.

## 2 Methods

A long‐standing collaboration between the Danish and Greenlandic healthcare system, including the transfer of patients in needs of specialized treatment to Danish hospitals (primarily Rigshospitalet University Hospital in Copenhagen), and the inclusion of all Greenlandic inhabitants in the Danish Civil Registration System (CRS), enabled us to follow the total Greenlandic and Danish population for neurological diagnoses in Danish as well as Greenlandic nationwide health registers as far back as 1987.

### 2.1 Study Population

The study cohort was identified by use of the Danish CRS and consisted of all subjects who lived in Denmark or Greenland at some time between the 1^st^ of January 1987 and 31st of December 2014, with a known place of birth (Pedersen et al. 2006) (*n* = 7,949,960). Since April 1968 and May 1972, respectively, the CRS has assigned every Danish and Greenlandic citizen, a unique personal identification number (ID). Linked to this ID number, the register contains information on, for example, sex, place of birth, parental identity, vital status, marital status, and residence (addresses in Danish and Greenlandic municipalities) (Pedersen et al. 2006).

### 2.2 Data Sources of Neurological Disorders

Information on neurological disorders was obtained from the Danish National Patient Registry (DNPR) and the Greenlandic National Patient Registry (GNPR). The DNPR contains records on all non‐psychiatric hospital admissions in Denmark since January 1977, including outpatient contacts since 1995 (Schmidt et al. 2015), whereas the GNPR includes information about inpatient contacts at Greenlandic hospitals since 1987 (Koch et al. 2011). Diagnoses in the GNPR and DNPR are classified according to the WHO International Classification of Diseases (ICD), using the 8th revision (ICD‐8) from 1977 to 1993 and the 10th revision (ICD‐10) since January 1994 (Schmidt et al. 2015). We obtained information about all registrations of dementia, PD, epilepsy, ischemic stroke, subarachnoid hemorrhage (SAH), intracerebral hemorrhage including other non‐traumatic intracranial hemorrhages (ICH), transient ischemic attack (TIA), meningitis, encephalitis including meningoencephalitis and encephalomyelitis, and other CNS infections according to the ICD codes listed in Table S1. Disorders were chosen if expected to be critical, primarily affecting adults, and to be common CNS disorders in the Danish population as described by Vestergaard et al. (Vestergaard et al. 2020), excluding mental disorders, cancers, injuries, and headache.

For reasons of comparison, we only included inpatients. We defined the date of diagnosis of a neurological disorder as the first date of an inpatient hospital contact with the disease in question (main and secondary diagnosis). Data from the GNPR was available until the beginning of 2015, the follow‐up period in the present study was therefore restricted to the period 1987 to 2014.

### 2.3 Risk Factors

Ethnicity (Inuit, Non‐Inuit), sex, and country of residence (Greenland or Denmark) were defined according to information in the CRS. We defined an Inuit as a person with at least one parent registered as born in Greenland. If there was no information on parental birthplace, ethnicity was defined according to the person's own place of birth.

### 2.4 Statistics

We estimated crude incidence rates (IRs) and prevalence in order to explore the absolute burden of neurological disorders in Greenland and the extent of the need for health services in the Greenlandic society. For comparison between rates in Greenland and Denmark, and to give the reader the possibility of comparing incidences with corresponding rates in other countries, we also calculated age‐standardized IRs.

Follow‐up for neurological diseases began on January 1, 1987 or birth, whichever occurred last, until death, emigration, or December 31, 2014, whichever came first. Individuals were allowed to have more than one neurological disorder but in the overall group of stroke comprising ischemic stroke, SAH, ICH and TIA, we only included the first incident diagnosis of either SAH, ICH, TIA or ischemic stroke. IRs per 100,000 person‐years of risk and corresponding 95% confidence intervals (CIs) were calculated for dementia, PD, epilepsy, ischemic stroke, ICH, SAH, TIA, meningitis, encephalitis, and other CNS infections using log linear Poisson‐regression for the combined period 1987 to 2014 and stratified by sex, ethnicity (Inuit and Non‐Inuit), country (Denmark (DK) and Greenland (GL)) and periods (1987–1994, 1995–2004, and 2005–2014).

For every study participant person‐years of risk was counted according to whether and when the person had residence in a Danish or a Greenlandic municipality. An incident case of a neurological disorders was counted as “Greenlandic” or “Danish” according to whether the person had residence in Greenland or Denmark at date of the first diagnosis of the disease in question.

Prevalence of dementia, PD, epilepsy and stroke (survivors) were calculated by counting the number of cases with the neurological disorder in question on December 31, 2014 divided by the size of the country's population the same date.

For the combined period 1987–2014, Greenlandic and Danish direct age‐standardized incidence rates (ASIR) were estimated for each of the neurological disorders using the WHO 2000–2025 population as standard (Ahmad et al. 2001; Naing 2000). The relative risks of neurological disorders in the Greenlandic population versus that in the Danish, were estimated by comparing ASIR for each of the neurological disorders in the Greenlandic population with the corresponding ASIR in the Danish population (ASIR_GL_/ASIR_DK_). ASIRs were furthermore stratified by sex and ethnicity.

### 2.5 Supplementary Analyses

To further explore the importance of ethnicity, we calculated the ratio of the observed numbers of each of the neurological disorders among Inuit in Greenland to the numbers expected if Inuit living in Greenland were at the same risk as Non‐Inuit living in Denmark (the majority of the Danish population), that is, Standardized Incidence Ratios (SIRs). The expected number was calculated as the sum of the age‐ (5‐years intervals), sex‐, and period‐specific (4‐years intervals) person‐years at risk in the Greenlandic Inuit population, multiplied by the corresponding IRs of the specific neurological disorder in the Danish Non‐Inuit population based on registration in the DNPR and GNPR from 1987 to 2014. Similar ratios were calculated for Inuit living in Denmark and Non‐Inuit living in Greenland using the Danish Non‐Inuit population as reference.

All estimates were calculated using the SAS Genmod procedure in SAS version 9.4.

## 3 Results

The study cohort consisted of 7,949,960 individuals who between 1987 and 2014 at some time had lived in Greenland or Denmark.

### 3.1 Crude Greenlandic IRs of Neurological Disorders

For the combined period 1987 to 2014 the crude IRs per 100,000 person‐years at risk for dementia, PD, epilepsy, meningitis, encephalitis, and other CNS infections in the Greenlandic population was IR_dementia _= 21.9 (95% CI: 19.7–24.3), IR_PD _= 8.35 (95% CI: 7.03–9.91), IR_epilepsy _= 98.6 (95% CI: 93.8–104), IR_meningitis _= 23.8 (95% CI: 21.5–26.3), IR_encephalitis _= 6.76 (95% CI: 5.58–8.17) and IR_cns_other _= 8.67 (95% CI: 7.33–10.3), respectively. The overall IR of stroke was 181 (95% CI: 174–188) with ischemic stroke accounting for more than 60% of these cases (IR_ischemic stroke _= 118 (95% CI: 113–124)) (Table 1).

**TABLE 1:** Crude incidence rates (IRs) and direct age‐standardized incidence rates (ASIRs) per 100,000 person‐years at risk for dementia, PD, epilepsy, ischemic stroke, SAH, ICH, TIA, meningitis, encephalitis, and other CNS infections, 1987–2014 in the Danish and Greenlandic populations according to country of residence.

	Greenlandic residence				Danish residence				ASIR_GL_/ASIR_DK_
	PYRS	Cases (*n*)	IR (95% CI) per 100,000	ASIR (95% CI) per 100,000	PYRS	Cases (*n*)	IR (95% CI) per 100,000	ASIR (95% CI) per 100,000	
Dementia	1569	343	21.9 (19.7–24.3)	34.0 (31.2–37.0)	149210	120691	80.9 (80.4–81.3)	38.1 (37.8–38.4)	0.89 (0.82–0.97)
PD	1570	131	8.35 (7.03–9.91)	11.6 (10.0–13.4)	149462	25322	16.9 (16.7–17.2)	9.20 (9.05–9.36)	1.26 (1.09–1.46)
Epilepsy	1550	1528	98.6 (93.8–104)	102 (96.9–107)	148321	85237	57.5 (57.1–57.9)	53.0 (52.6–53.3)	1.92 (1.83–2.02)
Stroke (all)^a^	1551	2804	181 (174–188)	236 (228–243)	147037	404292	275 (274–276)	169 (168–169)	1.40 (1.35–1.44)
Ischemic stroke	1559	1842	118 (113– 124)	156 (150–163)	147965	288314	195 (194–196)	114 (113–115)	1.37 (1.32–1.43)
SAH	1566	357	22.8 (20.5–25.3)	22.4 (20.2–24.9)	149429	18738	12.5 (12.4–12.7)	9.49 (9.34–9.65)	2.36 (2.12–2.62)
ICH	1568	420	26.8 (24.3–29.5)	32.0 (29.3–34.9)	149371	50553	33.8 (33.6–34.1)	20.8 (20.5–21.0)	1.54 (1.41–1.68)
TIA	1566	597	38.1 (35.2–41.3)	49.3 (46.0–52.9)	148769	105528	70.9 (70.5–71.4)	42.8 (42.5–43.2)	1.15 (1.07–1.24)
Encephalitis	1569	106	6.76 (5.58–8.17)	6.75 (5.58–8.16)	149428	8265	5.53 (5.41–5.65)	5.28 (5.16–5.40)	1.28 (1.05–1.55)
Meningitis	1565	372	23.8 (21.5–26.3)	23.5 (21.2–26.0)	149181	20930	14.0 (13.8–14.2)	15.3 (15.1–15.5)	1.53 (1.38–1.70)
Other CNS infections	1569	136	8.67 (7.33–10.3)	8.71 (7.36–10.3)	149480	9386	6.28 (6.15–6.41)	4.91 (4.80–5.02)	1.77 (1.50–2.10)

Abbreviations: ASIR, Age standardized incidence rate per 100,000 person‐years of risk, according to WHO standard population, IR, Incidence rates per 100,000 person‐years of risk; ASIR_GL_/ASIR_DK_, ratio between ASIRs in Greenland and Denmark; CI, confidence intervals; DK, Denmark; GL, Greenland; PD, Parkinson's Disease; PYRS, person‐years at risk in thousands; SAH, subarachnoid hemorrhage; TIA, transient ischemic attack; ICH, Intracerebral hemorrhage including other nontraumatic intracranial hemorrhages.

^a^ In stroke (all) only the first incident diagnosis of either SAH, ICH, TIA or ischemic stroke is included.

**FIGURE 1:**
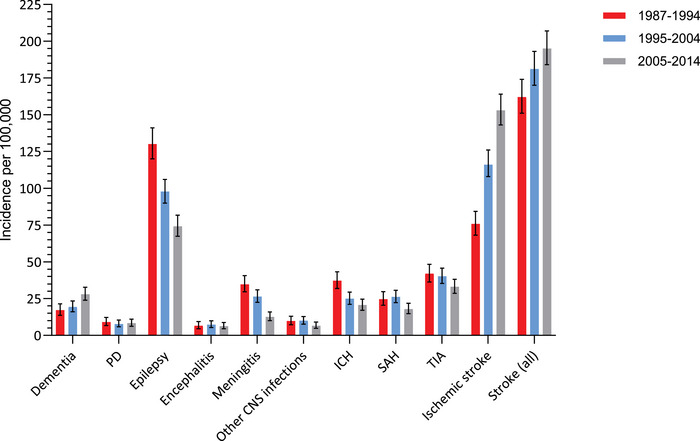
Crude incidence rates per 100,000 person‐years of risk of dementia, PD, epilepsy, encephalitis, meningitis, other CNS infections, SAH, ICH, TIA and ischemic stroke in the Greenlandic population in the periods 1987–1994, 1995–2004, and 2005–2014. Abbreviations: ICH, intracerebral hemorrhage including other nontraumatic intracranial hemorrhages; PD, Parkinson's disease; SAH, subarachnoid hemorrhage; TIA, transient ischemic attack. In stroke (all) only the first incident diagnosis of either SAH, ICH, TIA or ischemic stroke is included.

Crude IRs of PD, encephalitis and other CNS infections appeared to be stable over time, whereas a markedly increased in IR was observed for ischemic stroke from 1987–1994 to 2005–2014. A more modest increase was observed for dementia, especially from 1995–2004 to 2005–2014. Crude IRs of the remaining neurological disorders, however most obvious for epilepsy and meningitis, seem to decline over time, but CIs for the different follow‐up periods were often overlapping (Figure 1, Table S2).

### 3.2 Age‐Standardized Greenlandic IRs of Neurologic Disorders

ASIR for TIA (ASIR_TIA _= 49.3 (95% CI: 46.0–52.9)), dementia (ASIR_dementia _= 34.0 (95% CI: 31.2–37.0)) and especially for Ischemic stroke (ASIR_ischemic stroke _= 156 (95% CI:150–163), were higher than corresponding crude IRs, otherwise only minor changes were observed (Table 1).

ASIR of ischemic stroke and epilepsy were higher among Greenlandic males compared with Greenlandic females, whereas SAH was more common among females. Besides that, no obvious differences in sex‐specific Greenlandic ASIRs were observed (Figure 2a, Table S2).

**FIGURE 2a:**
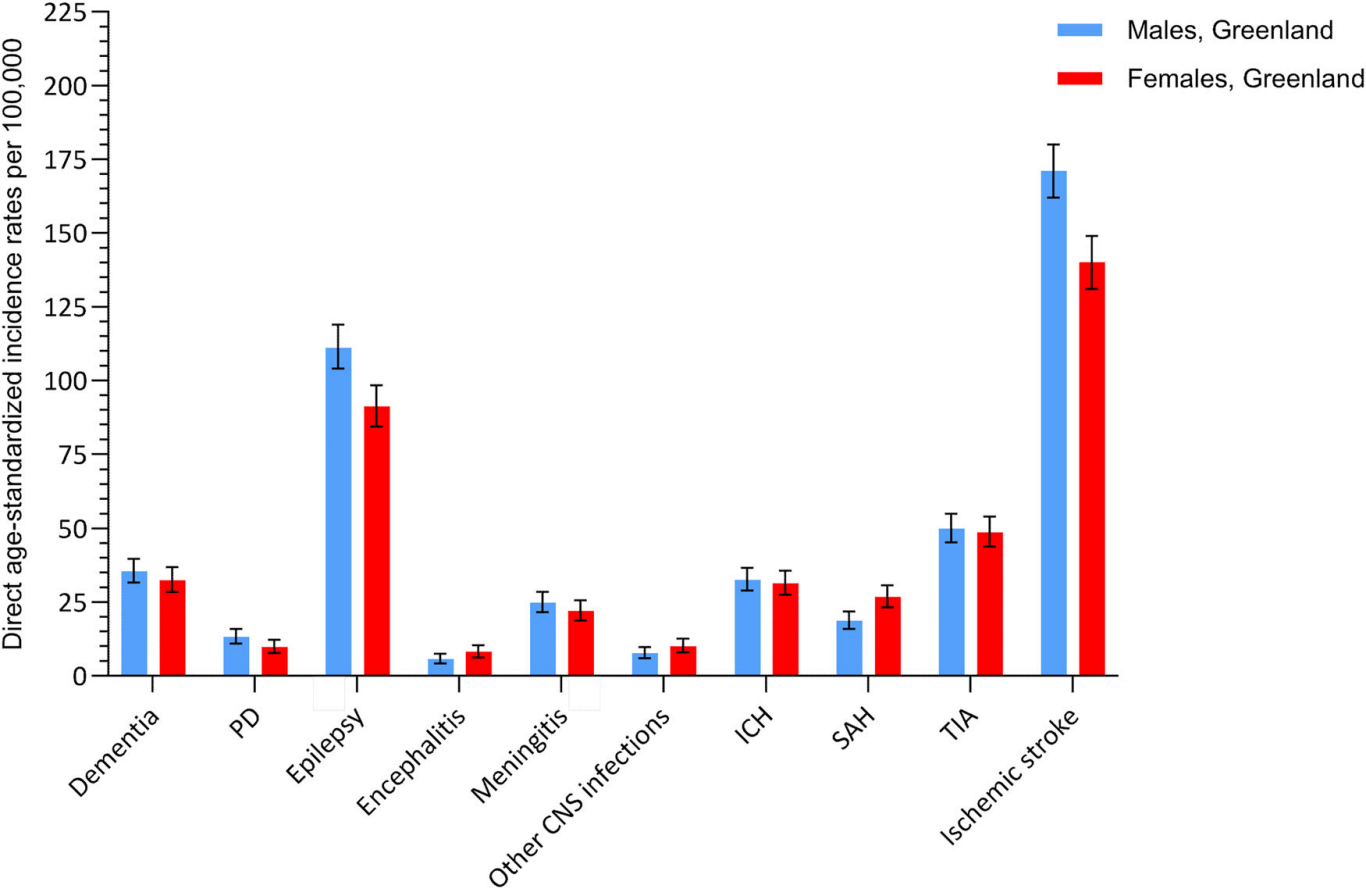
Direct age‐standardized incidence rates per 100,000 person‐years of risk of dementia, PD, epilepsy, encephalitis, meningitis, other CNS infections, SAH, ICH, TIA, and ischemic stroke among Greenlandic males and females 1987–2014. Abbreviations: SAH, Subarachnoid hemorrhage; TIA, transient ischemic attack; ICH, intracerebral hemorrhage including other nontraumatic intracranial hemorrhages; PD, Parkinson's disease. Direct age standardized incidence rates were calculated using the WHO 2000–2025 population as standard.

### 3.3 Age‐Standardized IRs of Neurologic Disorders According to Country of Residence and Sex

Apart from dementia, the Greenlandic population was generally at an elevated risk of the investigated neurological disorders compared with the Danish population, most pronounced for SAH (ASIR_GL_SAH_/ASIR_DK_SAH_ = 2.36 (95% CI: 2.12–2.62)) (Table 1). A similar pattern was seen for Greenlandic women compared with Danish women, whereas Greenlandic males had no elevated risk of PD, encephalitis and TIA, and generally less pronounced elevated risks of the other subtypes of stroke compared with Danish males (Figure 2b, Table S2).

**FIGURE 2b:**
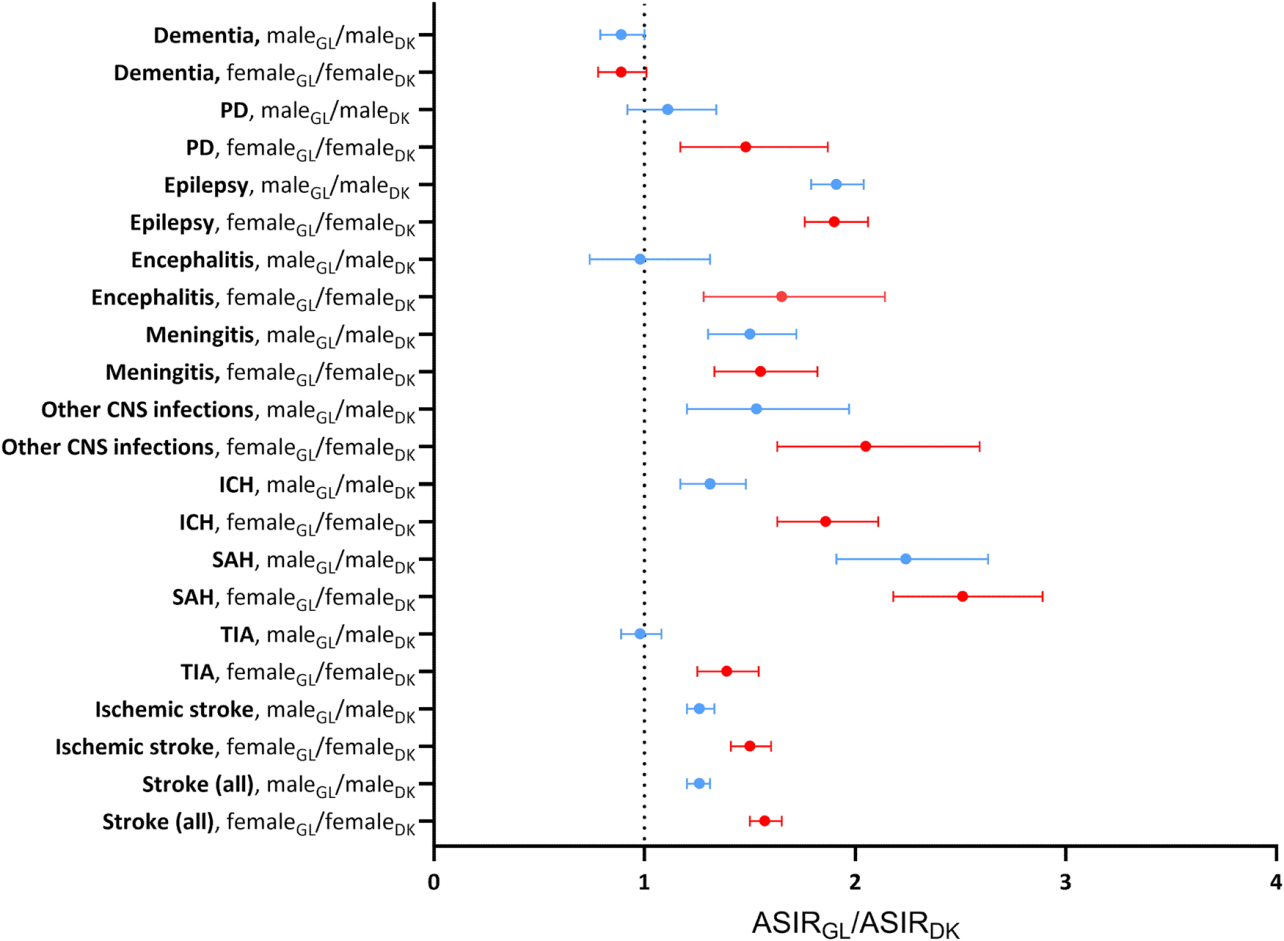
The ratio of age‐standardized incidence rates (ASIRs) of dementia, PD, epilepsy, encephalitis, meningitis, other CNS infections, SAH, ICH, TIA, and ischemic stroke among Greenlandic females and males, to the corresponding ASIRs among Danish females and males, respectively, 1987–2014. Abbriviations: ASIR_GL_/ASIR_DK_, direct age standardized incidence rate ratios comparing ASIRs in Greenland (GL) with corresponding ASIRs in Denmark (DK); ICH, intracerebral hemorrhage including other nontraumatic intracranial hemorrhages; PD, Parkinson's disease; SAH, subarachnoid hemorrhage; TIA, transient ischemic attack. In stroke (all) the only first incident diagnosis of either SAH, ICH, TIA or ischemic stroke is included. Age standardized incidence rates (ASIRs) were calculated using the WHO 2000–2025 population as standard.

### 3.4 Age‐Standardized IRs of Neurologic Disorders According to Country of Residence and Ethnicity

The ASIRs of epilepsy, stroke (all, and subtypes), and meningitis among Inuit living in Greenland were considerably higher than corresponding rates among Non‐Inuit in Greenland (the majority Danes). Whereas ASIRs for the remaining disorders were compatible or slightly higher among Inuit, but CIs were overlapping   (Figure 3a, Table S2). Inuit living in Greenland were at a lower risk of the overall group of strokes, ischemic stroke, SAH, ICH, PD and especially dementia (ASIR_GL_dementia_/ASIR_DK_dementia_ = 0.40 (95% CI: 0.35–0.46)), compared with Inuit living in Denmark, but at a slightly elevated risk of epilepsy (ASIR_GL_epilepsy_ /ASIR_DK_epilepsy_ = 1.11 (95% CI: 1.0–1.13)), although this estimate did not reach statistically significance. Country of residence did not seem to have an obvious impact on the risk of CNS infections among Inuit. (Figure 3b, Table S2).

**FIGURE 3a:**
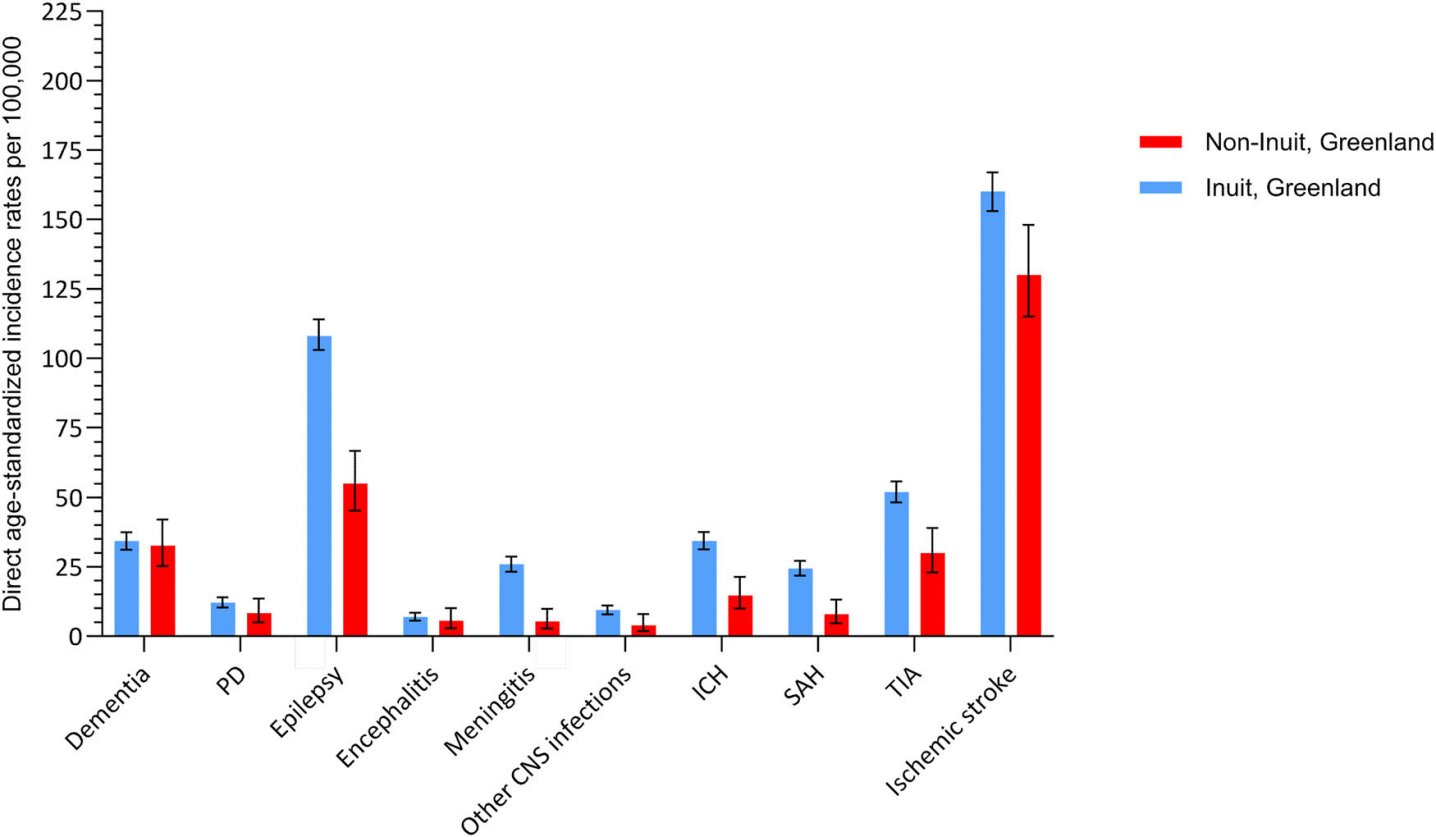
Direct age‐standardized incidence rates per 100,000 person‐years of risk of dementia, PD, epilepsy, encephalitis, meningitis, other CNS infections, SAH, ICH, TIA, and ischemic stroke, among Inuit and Non‐Inuit living in Greenland. Abbreviatons: ICH, intracerebral hemorrhage including other nontraumatic intracranial hemorrhages; SAH, subarachnoid hemorrhage; TIA, transient ischemic attack; PD, Parkinson's disease. Age‐standardized incidence rates were calculated using the WHO 2000–2025 population as standard.

**FIGURE 3b:**
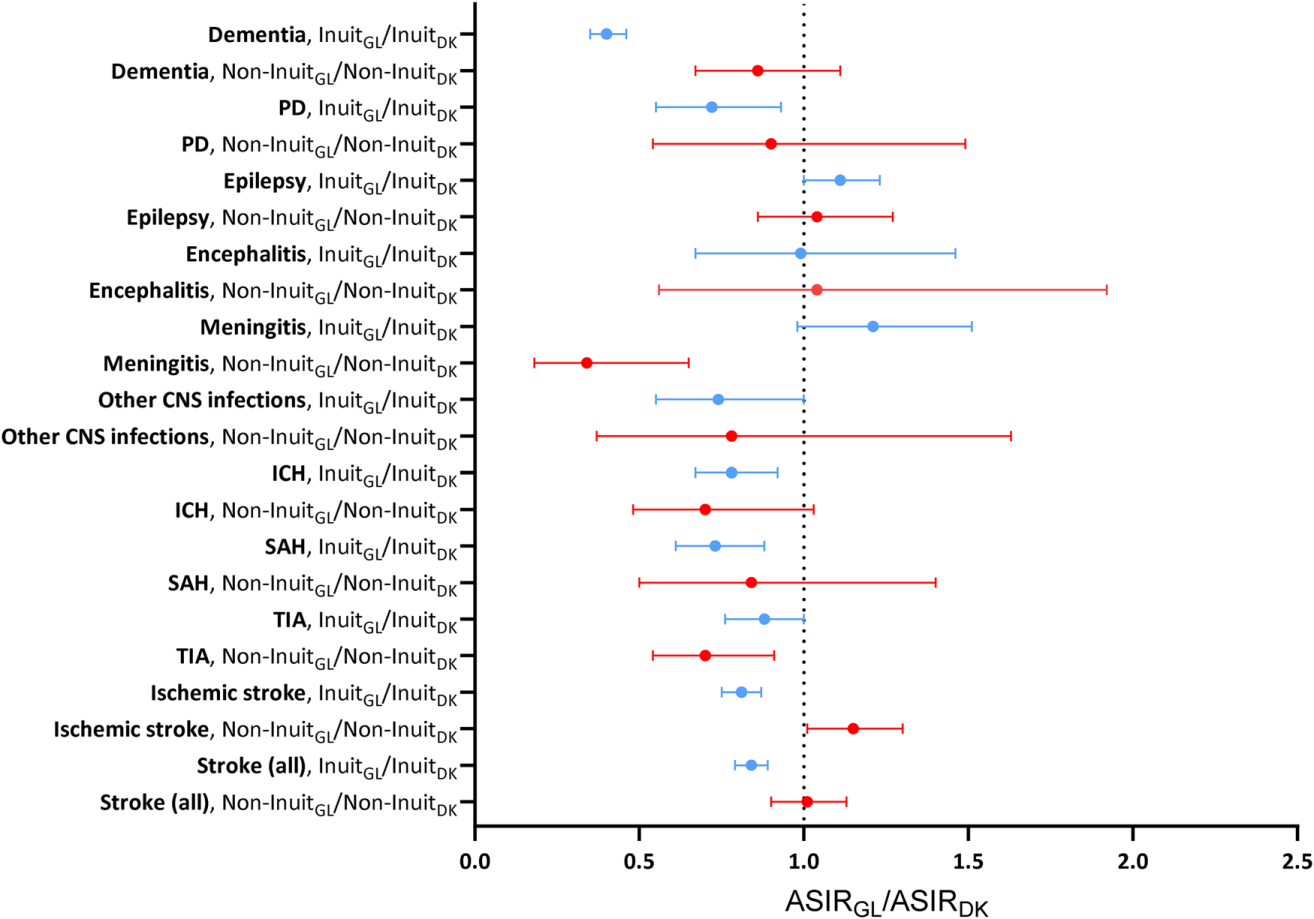
The ratio of age‐standardized incidence rates (ASIRs) of dementia, PD, epilepsy, encephalitis, meningitis, other CNS infections, SAH, ICH, TIA, and ischemic stroke, among Inuit and Non‐Inuit living in Greenland, to the corresponding ASIRs of neurological disorders among Inuit and Non‐Inuit living in Denmark, respectively, 1987–2014. Abbreviations: ASIR_GL_/ASIR_DK_, direct age standardized incidence rate ratios comparing ASIR in Greenland (GL) with corresponding ASIR in Denmark (DK); ICH, intracerebral hemorrhage including other nontraumatic intracranial hemorrhages; PD, Parkinson's disease; SAH, subarachnoid hemorrhage; TIA, transient ischemic attack. In stroke (all) only the first incident diagnosis of either SAH, ICH, TIA or ischemic stroke is included. Age standardized incidence rates (ASIR) were calculated using the WHO 2000–2025 population as standard.

### 3.5 Crude Prevalence of Neurological Disorders

By December 31, 2014, the prevalence of dementia, PD, epilepsy, and stroke (survivors) in the Greenlandic population was 168, 64, 1799, and 2059 per 100,000, respectively. The prevalence of epilepsy and stroke among Inuit in the Greenlandic population was almost twice as high as the corresponding prevalence among Non‐Inuit in the Greenlandic population. Dividing stroke into subtypes revealed that the very high prevalence of stroke among Inuit in Greenland was due to an ischemic stroke (Table 2).

**TABLE 2:** Crude prevalence per 100,000 of dementia, PD, epilepsy, ischemic stroke, SAH, ICH, and TIA, the 31st of December 2014 in the total Greenlandic population and according to ethnicity.

	Total Greenlandic population *n* = 55,909		Greenlandic Inuit population *n* = 50,724		Greenlandic Non‐Inuit population *n* = 5,185	
	Cases (*n*)	Prevalence per 100,000	Cases (*n*)	Prevalence per 100,000	Cases (*n*)	Prevalence per 100,000
Dementia^a^	94	168.1	—	—	—	—
PD^a^	36	64.39	—	—	—	—
Epilepsy	1006	1799	956	1885	50	964.3
Stroke (all)^b^	1151	2059	1086	2141	65	1254
Ischemic stroke	774	1384	729	1437	45	867.9
SAH	184	329.1	176	347.0	8	154.3
ICH	120	214.6	112	220.8	8	154.3
TIA	249	445.4	237	467.2	12	231.4

Abbreviations: ICH, intracerebral hemorrhage including other nontraumatic intracranial hemorrhages; PD, Parkinson's disease; SAH, subarachnoid hemorrhage; TIA, Transient ischemic attack.

^a^ To few cases among Non‐Inuit living in Greenland, accordingly ethnic specific prevalence for dementia and PD cannot be shown.

^b^ In stroke (all) only the first incident diagnosis of either SAH, ICH, TIA, or ischemic stroke is included.

### 3.6 Standardized Incidence Ratios of Neurological Disorders

Findings in the analysis using the Danish Non‐Inuit population as reference are compatible with previous observations concerning ethnicity and country of residence. Dementia stood out as Greenlandic Inuit living in Denmark compared with the Danish Non‐Inuit population, were at a 2.5–fold elevated risk of dementia SIR_dementia _= 2.49 (95% CI: 2.11–2.95), whereas no difference was observed for Inuit living in Greenland (SIR_dementia _= 1.09 (95% CI: 0.98–1.21)) (Table 3).

**TABLE 3:** Expected and observed number of neurological disorders and corresponding standard incidence ratios (SIRs) in the Danish and Greenlandic population, 1987–2014, according to ethnicity (Inuit, Non‐Inuit), using Non‐Inuit in Denmark as reference.

	Greenlandic residence			Danish residence		
	Obs.	Exp.^a^	SIR (95% CI)	Obs.	Exp.^a^	SIR (95% CI)
Dementia						
Ethnicity						
Inuit	323	296.49	1.09 (0.98–1.21)	136	54.60	2.49 (2.11–2.95)
Non‐Inuit	20	25.19	0.79 (0.51–1.23)	120555	120555.00	1.00 (0.99–1.01)^b^
PD						
Inuit	125	78.68	1.59 (1.33–1.89)	23	13.73	1.68 (1.11–2.52)
Non‐Inuit	6	9.31	0.64 (0.29–1.43)	25299	25299.00	1.00 (0.99–1.01)^b^
Epilepsy						
Inuit	1437	696.50	2.06 (1.96–2.17)	390	237.89	1.64 (1.48–1.81)
Non‐Inuit	91	91.47	0.99 (0.81–1.22)	84847	84847.00	1.00 (0.99–1.01)^b^
Ischemic stroke						
Inuit	1672	1100.10	1.52 (1.45–1.59)	481	225.63	2.13 (1.95–2.33)
Non‐Inuit	170	173.92	0.98 (0.84–1.14)	287833	287833.00	1.00 (1.00–1.00)^b^
SAH						
Inuit	334	121.99	2.74 (2.46–3.05)	131	37.06	3.53 (2.98–4.19)
Non‐Inuit	23	21.28	1.08 (0.72–1.63)	18607	18607.00	1.00 (0.99–1.01)^b^
ICH						
Inuit	394	224.54	1.75 (1.59–1.94)	116	51.68	2.24 (1.87–2.69)
Non‐Inuit	26	37.54	0.69 (0.47–1.02)	50437	50437.00	1.00 (0.99–1.01)^b^
TIA						
Inuit	545	450.47	1.21 (1.11–1.32)	160	102.09	1.57 (1.34–1.83)
Non‐Inuit	52	75.23	0.69 (0.53–0.91)	105368	105368.00	1.00 (0.99–1.01)^b^
Encephalitis						
Inuit	95	72.39	1.31 (1.07–1.60)	31	25.29	1.23 (0.86–1.74)
Non‐Inuit	11	9.72	1.13 (0.63–2.04)	8234	8234.00	1.00 (0.98–1.02)^b^
Meningitis						
Inuit	364	216.41	1.68 (1.52–1.86)	106	80.88	1.31 (1.08–1.59)
Non‐Inuit	8	22.62	0.35 (0.18–0.71)	20824	20824.00	1.00 (0.99–1.01)^b^
Other CNS infections						
Inuit	125	63.09	1.98 (1.66–2.36)	43	19.03	2.26 (1.68–3.05)
Non‐Inuit	11	10.40	1.06 (0.59–1.91)	9343	9343.00	1.00 (0.98–1.02)^b^

Abbreviations: CI, confidence interval; Exp, expected numbers; ICH, intracerebral hemorrhage including other nontraumatic intracranial hemorrhages; Obs, observed numbers; PD, Parkinson's disease; SAH, subarachnoid hemorrhage; SIR, standardized Incidence ratio; TIA, transient ischemic attack.

^a^Expected number of cases and SIR were calculated using Danish age‐, sex‐, and period‐specific rates based on information from the DNPR and GNPR for the period 1987–2014 using the Danish Non‐Inuit population as reference.

^b^Reference group.

## 4 Discussion

We observed high crude incidences and prevalence of epilepsy and ischemic stroke in the Greenlandic population, higher in Inuit than in non‐Inuit. Crude Greenlandic IRs of ischemic stroke and to a lesser degree dementia seem to increase over time, whereas IRs of epilepsy and meningitis appeared to decline.

Apart from dementia, ASIRs of the explored neurological disorders were higher in the Greenlandic population compared with corresponding rates in the Danish population and apparently most pronounced for women. Interestingly, Inuit living in Denmark appeared to be at a higher risk of PD, ischemic stroke, SAH, and ICH, and a markedly higher risk of dementia compared to Inuit living in Greenland.

### 4.1 Dementia and PD

We found a crude prevalence of dementia in 2014 (168 per 100,000) considerably lower than that predicted by the GBD group in the year 2016 (270 per 100,000) (GBD Dementia Collaborators 2019) and less than 40% of that reported in a Governmental survey of dementia from 2011, based on registrations done by the staff at nursing homes or institutions (The Greenlandic Goverment 2012). The crude prevalence of PD in our study (64 per 100,000) was likewise lower than previous estimates around 80 per 100,000 (GBD Parkinson's Disease Collaborators 2018; Wermuth et al. 2004). In addition, to hospital data Wermuth et al. used information from nursing homes, outpatient clinics and use of levodopa (Wermuth et al. 2004). In our study we only relied on hospital data, which seem to underestimate the burden of PD and especially dementia in Greenlandic population.

We observed a tendency toward an increase in the crude IRs of dementia in the Greenlandic population especially from 1995 to 2014, which is compatible with trends seen in the Danish population from 1995 to 2003 (Taudorf et al. 2019). The incidence of dementia in Denmark stabilized around 2003, but has declined since 2010 presumable due to better living conditions including of better education and management of risk factors for dementia (Taudorf et al. 2019). Cohort studies from the UK and USA have likewise observed a decline in the incidence of dementia although rates of decline differed substantially between studies (Mukadam et al. 2024). Globally, the age‐standardized incidence of dementia has not yet declined (Xu et al. 2025). Of concern is a 2.5–fold higher risk of dementia among Inuit living in Denmark compared with Inuit in Greenland. The mechanism behind this is unknown, but one could speculate that adaption of a less favorable lifestyle in Denmark in combination with genetic factors (Hegele et al. 1997) could be of importance. However, easier access to hospitals and dementia clinics in Denmark relative to Greenland leading to a higher rate of diagnosed cases may also contribute to this observation.

### 4.2 Epilepsy and CNS Infections

Epilepsy has been considered to be more frequent in Greenland than in Denmark (Blichfeldt et al. 2004). Nevertheless it surprising, that we found a crude prevalence of epilepsy in the Greenlandic population considerably higher than previous estimates for Greenland (Blichfeldt et al. 2004; GBD Epilepsy Collaborators 2019; Olesen 1996) and for Denmark (Christensen et al. 2024). It is difficult to explain this discrepancy. Diagnostic misclassification between epilepsy and other disorders with seizures might contribute to the high incidence and prevalence of epilepsy in the Greenlandic population. However, the fact that Inuit living in Denmark “only” had a slightly lower ASIR of epilepsy compared with Inuit living in Greenland speaks against diagnostic misclassification due, for example, to limited access to specialized medical staff in Greenland should be the only explanation.

We observed a decline in the crude IRs of epilepsy over time, which also have been observed among other indigenous populations (Hernandez‐Ronquillo et al. 2018). Better public health care, including good prenatal care, improved treatment of infections and cardiovascular diseases as well a better prevention of infections and head injury may contribute to this tendency (Hernandez‐Ronquillo et al. 2018). However, prevalent Greenlandic cases of epilepsy might have been registered as new incident cases in the beginning of the study period as the GNPR was “only” established in 1987. This could explain the very high incidence in the period 1987–1994. The decline in the crude IR of epilepsy did however continue even after 1994.

The crude IRs of meningitis and encephalitis estimated in the present study are close to corresponding rates found by Nordholm et al. for the combined period 1990–2012 (Nordholm et al. 2017). We furthermore observed declining crude IRs of meningitis, presumably associated with the beforementioned improved public health care in Greenland, but compatible ASIRs of CNS infections among Inuit living in Greenland and Denmark in spite of different living conditions, indicating that genetic factors may also play a role in the susceptibility to CNS infections among Inuit (Nordholm et al. 2017).

### 4.3 Stroke

The overall crude IRs and ASIRs of stroke in our study for the combined period 1987–2014 are higher than corresponding rates calculated by Bjorn‐Mortensen et al. from 2011 to 2012 (Bjorn‐Mortensen et al. 2013) but lower than those reported by Larsen et al. for the period 2005–2014 (Larsenet al. 2024). However, Bjorn‐Mortensen et al. (Bjorn‐Mortensen et al. 2013) only included validated stroke diagnoses (excluding TIA), and only for persons who survived stroke and subsequently could be transferred to Nuuk for evaluation. Larsen et al. used a broader definition of stroke, for example, including ill‐defined cerebrovascular disease as well as sequelae (Larsen et al. 2024) and furthermore added information from outpatient contacts for the period 2013–2017.

Our study is in line with findings by Bjorn‐Mortensen et al. who observed that the majority of stroke cases were due to ischemic stroke, and that the incidence of SAH were approximately three times as high as that in the Danish population (Bjorn‐Mortensen et al. 2013). Despite deviation in diagnostic criteria, we observed an increase in the crude overall IRs of stroke over time compatible with that observed by Larsen et al. (Larsen et al. 2024), probably associated with the observed raise in life expectancy in the Greenlandic population (data.worldbank.org). This is in contrast to the decline in the age‐standardized IRs of ischemic stroke observed in Western European countries from 1990 to 2021 (Li et al. 2024).

The Greenlandic population appeared to be at a 40% higher risk of stroke compared with the Danish population. Greenlandic females were generally at a higher risk of stroke compared with Danish females, which is in line with previous findings (Bjorn‐Mortensen et al. 2013; Truelsen et al. 2006), whereas the corresponding rates among Greenlandic males were less elevated and for TIA even similar to corresponding rates among Danish males. A sligthly higher proportion in the 2000s of Greenlandic female smokers than male smokers especially in the oldest age groups might contribute to this observation (Bjerregaard et al. 2008).

Inuit living in Denmark were overall at a higher risk of stroke compared with Inuit in Greenland. Bjerregaard et al. found that Inuit migrants in Denmark had significantly higher blood pressure than Inuit living in Greenland and suggested that this could be caused by blood pressure of the Inuit being responsive to certain factors related to the modern western way of life in Denmark (Bjerregaard et al. 2002), factors which may also be relevant for stroke.

In the present study, we included the ICD10 code for unspecific stroke (I64) in the definition of ischemic stroke, as more than 2/3 of these patients according to validation studies of stroke diagnoses registered in the DNPR, should have had an ischemic stroke (Krarup et al. 2007; Johnsen et al. 2002). The ICD8 codes 43601 and 43690 for unspecific stroke (apoplexy with and without hypertension) have to our knowledge not been validated and were therefore not at first included in the definition of ischemic stroke. However, we observed a very sharp increase in the Greenlandic crude IRs of ischemic stroke coinciding with the transition from ICD8 to ICD10 codes. By including the ICD8 codes for unspecific apoplexy, this sharp increase was reduced considerably (data not shown).

### 4.4 The GBD Studies

In the present study the crude IRs for the combined period 1987‐2014 for the overall group of strokes and meningitis, and the crude prevalence for epilepsy in 2014 were 1.2, 3.4 and 5.3–fold higher, respectively, than corresponding estimates based on counts in 2016 from the GBD studies (GBD Epilepsy Collaborators 2019; GBD Meningitis Collaborators 2018; GBD Stroke Collaborators 2019). On the other hand the crude prevalence predicted by the GBD studies focusing on PD and dementia, respectively, were 1.3 (GBD Parkinson's Disease Collaborators 2018) and 1.6 (GBD Dementia Collaborators 2019) fold higher than our estimates. It is difficult to explain the observed discrepancies. The codes used for epilepsy in our study is exactly the same as those used in the GBD study and for many of the other disorders, the selected ICD codes were overlapping but not identical. As noted previously, data in many regions of the world, including Greenland are sparse; accordingly some of the rates modeled by the GBD studies should be interpreted with caution.

### 4.5 Strength

To our knowledge, this is the first nationwide cohort study to estimate IRs, prevalence and relative risks of a broad group of neurological disorders in the Greenlandic population according to ethnicity and sex, using the Danish population as reference. Due to detailed information on country of residence, family relations and place of birth we were furthermore able to evaluate the importance of environmental exposures (living in Greenland or Denmark) and ethnicity (Inuit, Non‐Inuit) in the etiology of the studied neurological disorders by addressing disease pattern among Inuit who had migrated to Denmark. In this register‐based study, including all residents in Denmark and Greenland from 1987 to 2014, selection bias and loss to follow‐up should be minimal.

### 4.6 Limitations

We would have preferred to follow the Greenlandic population up to 2024, but updated data from the GNPR is not available at the moment. The results may therefore not reflect the current disease burden in Greenland. Nevertheless, the presented estimates based on a follow‐up period of almost 30 years provides us with the possibility of predicting the future epidemiological profile of a broad group of neurological disorders in the Greenlandic population. At the moment these data are the most recent data available for future resource planning in the Greenlandic healthcare system in the field of a broad group of neurological disorders.

The validity of most of the diagnoses in GNPR, apart from the cardiovascular diagnoses (Tvermosegaard et al. 2018), have furthermore not been evaluated yet, but the GNPR has overall been found to applicable for scientific purposes with a high completeness (Koch et al. 2011). However, due to the lack of specialist doctors in Greenland and the special geographic condition of Greenland which may hinder access to healthcare for many people living in remote settlements, the risk of misdiagnosis and underdiagnosis is probably higher in Greenland than in for example, Denmark (Koch et al. 2011).

In addition, overlapping symptoms can be a diagnostic challenge and render the differentiation between two neurological disorders. Due to lack of neurologists at Greenlandic hospitals this issue could be more pronounced in the GNPR. As mentioned previously, the very high incidence of epilepsy might partly be contributable to a confusion between the symptoms of epilepsy and seizures.

Despite severe challenges in recruitment of specialized healthcare personnel, a high frequent of staff turnover in the Greenlandic health care system and limited access to hospital treatment for Greenlanders living in remote settlements, we cannot rule out that the increase in IRs over time of for example, ischemic stroke may reflect improved diagnostic possibilities and capabilities in the Greenlandic Health care system (Sundhedskommissionen Midtvejsrapport 2020).

The GNPR does not include information on outpatient contacts, and in combination with no data on use of medication one would expect that for some of the neurological disorders examined in the present study the disease burden could be underestimated.

When comparing crude IRs and prevalence as well as ASIRs between Inuit living in Greenland and Denmark, respectively, one should be aware of a much easier access to hospitals and specialized treatment in Denmark. In addition, Inuit migrating to Denmark may not be representative for the total population of Inuit in Greenland. People may migrate for social, economic, or health‐related reasons thus the comparison of the two populations, must be made with caution (Moustgaard et al. 2005).

Other issues could be the transition from ICD8 codes to ICD10 codes in 1993. This could lead to non‐real apparent changes in IRs, as ICD8 and ICD10 codes do not always coincide, i.e. one‐to‐one mapping can be difficult. We tried to select overlapping ICD8 and ICD10 codes for the different neurological disorders according to previous studies and our own discretion. To evaluate the chosen codes, we plotted biennial crude IRs of all the neurological disorders from 1987 to 2014. The curves revealed a surprisingly high increase in the IR of ischemic stroke in the period 1993–1996 which made us revise the ICD8 codes for ischemic stroke as describe previously.

We cannot rule out that prevalent cases of neurological disorders might have been registered as incident cases in the beginning of the study period leading to too high IRs in the first years after 1987 in the Greenlandic population. As mentioned previously this could be an issue for epilepsy.

We did not include the length of duration of stay in Denmark among Inuit, nor age or year of migration. Thus, we did not have the opportunity to estimate the importance of duration and timing of environmental exposures on the risk of neurological disorders among Inuit living in Denmark.

## 5 Conclusion

Our study emphasizes that stroke, especially ischemic stroke, and epilepsy are important causes of morbidity in Greenland and suggests that dementia may become a challenge to the Greenlandic health care system in the future. Of concern is also the general higher risk of neurological disorders (apart from dementia) in the Greenlandic population compared with the Danish and that the incidence of ischemic stroke in contrast to other Western European countries still seem to be increasing. Hopefully, our study can provide the Greenlandic health care system with data for the use in the efforts to limit the future burden of neurological disorders in the country. To expand the knowledge on disease mechanisms and possible modifiable risk factors, the impact of ethnic and environmental factors on the risk of neurological disorders should be further explored. In particular, the mechanism behind the markedly increased risk of dementia among Inuit living in Denmark should be examined.

## Author Contributions

**Nete Munk Nielsen**: funding acquisition, writing – original draft, investigation, conceptualization, methodology, visualization, project administration, data curation, resources. **Mikael Andersson**: data curation, supervision, formal analysis, software, validation, methodology, writing – review and editing. **Melinda Magyari**: writing – review and editing, data curation, supervision, validation. **Nils Koch‐henriksen**: data curation, writing – review and editing, supervision, validation. **Egon Stenager**: supervision, data curation, conceptualization, funding acquisition, validation, writing – review and editing. **Anders Koch**: data curation, supervision, writing – review and editing, conceptualization, funding acquisition, validation.

## Funding

The study was funded by Lilly & Herbert Hansen's Foundation, the Greenland Research Council and A. P. Moller's Foundation (Fonden til Lægevidenskabelig Fremme).

## Conflicts of Interest

NMN, MA, NKH, ES and AK have nothing to disclose. MM has served on scientific advisory board as consultant for, received support for congress participation or speaker honoraria from Biogen, Sanofi, Roche, Novartis, Merck, Alexion, Bristol Myers Squibb. The Danish MS Registry received research support from Biogen, Genzyme, Roche, Merck, Novartis.

## Ethics Statement

The study was approved by the commission for Scientific Research in Greenland (Approval No. 2019 – 16080) and the Department of Data Protection and Information Security at Statens Serum Institut (Approval No. 21/00254).

## Supporting information




**Supporting Materials**: brb371086‐sup‐0001‐tables.docx

## Data Availability

Data sharing not appliccable to this article as no new datasets were generated or analysed during the current study.
