# Supplementary material for: Severe Neurological Disorders in the Greenlandic Population: A Nationwide Register‐Based Study
Source: Brain Behav. 2025 Dec 22;15(12):e71086. doi: 10.1002/brb3.71086 (PMC12723079; doi:10.1002/brb3.71086)
Supplement: Supplementary file 1 — Supporting Materials: brb371086‐sup‐0001‐tables.docx [file BRB3-15-e71086-s001.docx]

**Table S1**: ICD8 and ICD10 codes for neurological disorders.

|  | |  |  |
| --- | --- | --- | --- |
|  | |  |  |
| Disorders | | ICD8 | ICD10 |
|  | |  |  |
|  | |  |  |
| Parkinson’s Disease **(PD)** | | 342 | G20 |
|  | |  |  |
| Epilepsy | | 345 | G40, G41 |
|  | |  |  |
| Dementia | | 290, 29309, 29319 | F00, F01, F020, F039, G30, G318, G319 |
|  | |  |  |
| Stroke | |  |  |
|  | Ischemic stroke | 433, 434, 43601, 43690 | I63, I64 |
|  | Subarachnoid hemorrhage **(SAH)** | 430 | I60 |
|  | Intracerebral hemorrhage incl. other nontraumatic intracranial hemorrhages **(ICH)** | 431 | I61, I62 |
|  | Transient ischemic attack **(TIA)** | 435 | G45 |
|  | Stroke (all) | 430, 431, 433-435, 43601, 43690 | G45, I60-I64 |
|  | |  |  |
| Meningitis | |  |  |
|  | Bakterial meningitis | 03609, 32009, 32019, 32080 | A022C, A229C, A321A, A390, A514B, A548D, G00, G01, |
|  | Viral meningitis | 045, 07501, 07929 | A87, B003, B010, B021, B051, B060B, B261, G020, |
|  | Other types of meningitis  (some may be non-infectious) | 32089, 32090, 32099 | B375, B384, B451A, G021, G028, G03, |
|  | |  |  |
| Encephalitis* | |  |  |
|  | Bacterial encephalitis | 02701 | G042, G050, A321B |
|  | Viral encephalitis | 05201, 05302, 05403, 05501, 05601, 07202, 062-065 | A83-A86, B004, B011, B020, B050, B060A, B060C, B262, G051, P352A |
|  | Other types of encephalitis  (some may be non-infectious) | 32091, 32092, 32093, 323 | B582, G040, G041, G048, G049, G052, G058 |
|  |  |  |  |
| Other Central Nervous System (CNS) infections | |  |  |
|  | Other viral CNS disorders | 046, 071, 07982, 33319, 33399 | A81, A82, A88, A89, B022 |
|  | Abscess of the CNS | 322 | A066, E236A, G06, G07 |
|  | TB of the nervous system | 013 | A17 |
|  | Poliomyelitis | 040-043 | A80 |
|  | Neurosyphilis | 09049,094 | A504, A521, A522, A523 |
|  |  |  |  |

CNS= Central nervous system, TB=Tuberculosis, *incl. myelitis/encephalomyelitis,

|  | |  |  |  |  |  |  |  |  |  |  |  |  |
| --- | --- | --- | --- | --- | --- | --- | --- | --- | --- | --- | --- | --- | --- |
|  | |  | Greenlandic residence | | | |  | Danish residence | | | |  | ASIR_GL_/ASIR_DK_ |
|  | |  |  |  |  |  |  |  |  |  |  |  |  |
|  | |  |  |  |  |  |  |  |  |  |  |  |  |
|  | |  | PYRS | Cases | IR (95% CI)  per 100,000 | ASIR (95% CI)  per 100,000 |  | PYRS | Cases | IR (95% CI)  per 100,000 | ASIR (95% CI)  per 100,000 |  |  |
|  | |  |  |  |  |  |  |  |  |  |  |  |  |
|  | |  |  |  |  |  |  |  |  |  |  |  |  |
| **Dementia** | |  | 1569 | 343 | 21.9 (19.7-24.3) | 34.0 (31.2-37.0) |  | 149210 | 120691 | 80.9 (80.4-81.3) | 38.1 (37.8-38.4) |  | 0.89 (0.82-0.97) |
| Sex | |  |  |  |  |  |  |  |  |  |  |  |  |
|  | Males |  | 837 | 163 | 19.5 (16.7-22.7) | 35.4 (31.6-39.7) |  | 73800 | 48189 | 65.3 (64.7-65.9) | 39.8 (39.3-40.3) |  | 0.89 (0.79-1.00) |
|  | Females |  | 731 | 180 | 24.6 (21.3-28.5) | 32.4 (28.5-36.8) |  | 75409 | 72502 | 96.1 (95.4-96.8) | 36.4 (36.0-36.9) |  | 0.89 (0.78-1.01) |
| Ethnicity | |  |  |  |  |  |  |  |  |  |  |  |  |
|  | Inuit |  | 1385 | 323 | 23.3 (20.9-26.0) | 34.2 (31.1-37.4) |  | 503 | 136 | 27.0 (22.9-32.0) | 85.6 (77.9-94.1) |  | 0.40 (0.35-0.46) |
|  | Non-inuit |  | 184 | 20 | 10.9 (7.01-16.9) | 32.6 (25.3-42.0) |  | 148707 | 120555 | 81.1 (80.6-81.5) | 37.9 (37.6-38.3) |  | 0.86 (0.67-1.11) |
|  |  |  |  |  |  |  |  |  |  |  |  |  |  |
|  | |  |  |  |  |  |  |  |  |  |  |  |  |
| **PD** | |  | 1570 | 131 | 8.35 (7.03-9.91) | 11.6 (10.0-13.4) |  | 149462 | 25322 | 16.9 (16.7-17.2) | 9.20 (9.05-9.36) |  | 1.26 (1.09-1.46) |
| Sex | |  |  |  |  |  |  |  |  |  |  |  |  |
|  | Males |  | 838 | 73 | 8.72 (6.93-11.0) | 13.2 (11.0-15.9) |  | 73883 | 14316 | 19.4 (19.1-19.7) | 11.9 (11.6-12.1) |  | 1.11 (0.92-1.34) |
|  | Females |  | 732 | 58 | 7.92 (6.13-10.2) | 9.71 (7.69-12.2) |  | 75579 | 11006 | 14.6 (14.3-14.8) | 6.57 (6.39-6.76) |  | 1.48 (1.17-1.87) |
| Ethnicity | |  |  |  |  |  |  |  |  |  |  |  |  |
|  | Inuit |  | 1386 | 125 | 9.02 (7.57-10.8) | 12.0 (10.3-14.0) |  | 503 | 23 | 4.57 (3.04-6.87) | 16.8 (13.6-20.8) |  | 0.72 (0.55-0.93) |
|  | Non-inuit |  | 184 | 6 | 3.26 (1.46-7.26) | 8.24 (4.98-13.6) |  | 148959 | 25299 | 17.0 (16.8-17.2) | 9.18 (9.02-9.33) |  | 0.90 (0.54-1.49) |
|  |  |  |  |  |  |  |  |  |  |  |  |  |  |
|  |  |  |  |  |  |  |  |  |  |  |  |  |  |
| **Epilepsy** | |  | 1550 | 1528 | 98.6 (93.8-104) | 102 (96.9-107) |  | 148321 | 85237 | 57.5 (57.1-57.9) | 53.0 (52.6-53.3) |  | 1.92 (1.83-2.02) |
| Sex | |  |  |  |  |  |  |  |  |  |  |  |  |
|  | Males |  | 827 | 879 | 106 (99.5-114) | 111 (104-119) |  | 73293 | 45908 | 62.6 (62.1-63.2) | 58.2 (57.6-58.7) |  | 1.91 (1.79-2.04) |
|  | Females |  | 723 | 649 | 89.7 (83.1-96.9) | 91.1 (84.4-98.4) |  | 75028 | 39329 | 52.4 (51.9-52.9) | 47.9 (47.4-48.4) |  | 1.90 (1.76-2.06) |
| Ethnicity | |  |  |  |  |  |  |  |  |  |  |  |  |
|  | Inuit |  | 1367 | 1437 | 105 (99.8-111) | 108 (103-114) |  | 497 | 390 | 78.5 (71.1-86.7) | 97.6 (89.3-107) |  | 1.11 (1.00-1.23) |
|  | Non-inuit |  | 183 | 91 | 49.8 (40.5-61.1) | 55.0 (45.2-66.8) |  | 147825 | 84847 | 57.4 (57.0-57.8) | 52.8 (52.4-53.2) |  | 1.04 (0.86-1.27) |
|  |  |  |  |  |  |  |  |  |  |  |  |  |  |
|  |  |  |  |  |  |  |  |  |  |  |  |  |  |
| **Ischemic stroke** | |  | 1559 | 1842 | 118 (113-124) | 156 (150-163) |  | 147965 | 288314 | 195 (194-196) | 114 (113-115) |  | 1.37 (1.32-1.43) |
| Sex | |  |  |  |  |  |  |  |  |  |  |  |  |
|  | Males |  | 832 | 1023 | 123 (116-131) | 171 (162-180) |  | 73084 | 146330 | 200 (199-201) | 135 (135-136) |  | 1.26 (1.20-1.33) |
|  | Females |  | 727 | 819 | 113 (105-121) | 140 (131-149) |  | 74882 | 141984 | 190 (189-191) | 93.2 (92.5-93.9) |  | 1.50 (1.41-1.60) |
| Ethnicity | |  |  |  |  |  |  |  |  |  |  |  |  |
|  | Inuit |  | 1376 | 1672 | 121 (116-127) | 160 (153-167) |  | 500 | 481 | 96.1 (87.9-105) | 197 (185-210) |  | 0.81 (0.75-0.87) |
|  | Non-Inuit |  | 183 | 170 | 92.8 (79.9-108) | 130 (115-148) |  | 147465 | 287833 | 195 (194-196) | 114 (113-114) |  | 1.15 (1.01-1.30) |
|  |  |  |  |  |  |  |  |  |  |  |  |  |  |

**Table S2:** Crude incidence rates (IRs) and direct age-standardized rates (ASIRs) per 100,000 person-years at risk for dementia, PD, epilepsy, meningitis, encephalitis, other CNS infections, SAH, ICH, TIA and ischemic stroke in the Greenlandic and Danish population, 1987-2014 according to country of residence, sex and ethnicity

**Supplementary table 2 continued**

|  | |  |  | | | |  |  | | | |  |  |
| --- | --- | --- | --- | --- | --- | --- | --- | --- | --- | --- | --- | --- | --- |
|  | |  | Greenlandic residence | | | |  | Danish residence | | | |  | ASIR_GL_/ASIR_DK_ |
|  | |  |  |  |  |  |  |  |  |  |  |  |  |
|  | |  |  |  |  |  |  |  |  |  |  |  |  |
|  | |  | PYRS | Cases | IR (95% CI)  per 100,000 | ASIR (95% CI)  per 100,000 |  | PYRS | Cases | IR (95% CI)  per 100,000 | ASIR (95% CI)  per 100,000 |  |  |
|  | |  |  |  |  |  |  |  |  |  |  |  |  |
|  | |  |  |  |  |  |  |  |  |  |  |  |  |
| **SAH** | |  | 1566 | 357 | 22.8 (20.5-25.3) | 22.4 (20.2-24.9) |  | 149429 | 18738 | 12.5 (12.4-12.7) | 9.49 (9.34-9.65) |  | 2.36 (2.12-2.62) |
| Sex | |  |  |  |  |  |  |  |  |  |  |  |  |
|  | Males |  | 836 | 165 | 19.7 (16.9-23.0) | 18.6 (15.9-21.8) |  | 73878 | 7825 | 10.6 (10.4-10.8) | 8.32 (8.12-8.53) |  | 2.24 (1.91-2.63) |
|  | Females |  | 730 | 192 | 26.3 (22.8-30.3) | 26.7 (23.2-30.7) |  | 75552 | 10913 | 14.4 (14.2-14.7) | 10.6 (10.4-10.9) |  | 2.51 (2.18-2.89) |
| Ethnicity | |  |  |  |  |  |  |  |  |  |  |  |  |
|  | Inuit |  | 1382 | 334 | 24.2 (21.7-26.9) | 24.3 (21.8-27.1) |  | 502 | 131 | 26.1 (22.0-30.9) | 33.2 (28.5-38.7) |  | 0.73 (0.61-0.88) |
|  | Non-inuit |  | 184 | 23 | 12.5 (8.31-18.8) | 7.90 (4.72-13.2) |  | 148927 | 18607 | 12.5 (12.3-12.7) | 9.41 (9.26-9.57) |  | 0.84 (0.50-1.40) |
|  |  |  |  |  |  |  |  |  |  |  |  |  |  |
|  |  |  |  |  |  |  |  |  |  |  |  |  |  |
| **ICH** | |  | 1568 | 420 | 26.8 (24.3-29.5) | 32.0 (29.3-34.9) |  | 149371 | 50553 | 33.8 (33.6-34.1) | 20.8 (20.5-21.0) |  | 1.54 (1.41-1.68) |
| Sex | |  |  |  |  |  |  |  |  |  |  |  |  |
|  | Males |  | 837 | 224 | 26.8 (23.5-30.5) | 32.5 (28.9-36.6) |  | 73824 | 26615 | 36.1 (35.6-36.5) | 24.8 (24.4-25.1) |  | 1.31 (1.17-1.48) |
|  | Females |  | 731 | 196 | 26.8 (23.3-30.8) | 31.3 (27.5-35.7) |  | 75547 | 23938 | 31.7 (31.3-32.1) | 16.9 (16.6-17.2) |  | 1.86 (1.63-2.11) |
| Ethnicity | |  |  |  |  |  |  |  |  |  |  |  |  |
|  | Inuit |  | 1384 | 394 | 28.5 (25.8-31.4) | 34.3 (31.3-37.5) |  | 503 | 116 | 23.1 (19.2-27.7) | 43.8 (38.4-50.0) |  | 0.78 (0.67-0.92) |
|  | Non-inuit |  | 184 | 26 | 14.1 (9.62-20.8) | 14.6 (9.98-21.3) |  | 148868 | 50437 | 33.9 (33.6-34.2) | 20.7 (20.5-20.9) |  | 0.70 (0.48-1.03) |
|  |  |  |  |  |  |  |  |  |  |  |  |  |  |
|  |  |  |  |  |  |  |  |  |  |  |  |  |  |
| **TIA** | |  | 1566 | 597 | 38.1 (35.2-41.3) | 49.3 (46.0-52.9) |  | 148769 | 105528 | 70.9 (70.5-71.4) | 42.8 (42.5-43.2) |  | 1.15 (1.07-1.24) |
| Sex | |  |  |  |  |  |  |  |  |  |  |  |  |
|  | Males |  | 836 | 314 | 37.6 (33.6-42.0) | 49.9 (45.3-54.9) |  | 73517 | 55424 | 75.4 (74.8-76.0) | 50.8 (50.2-51.3) |  | 0.98 (0.89-1.08) |
|  | Females |  | 730 | 283 | 38.8 (34.5-43.6) | 48.6 (43.8-54.0) |  | 75252 | 50104 | 66.6 (66.0-67.2) | 35.1 (34.7-35.5) |  | 1.39 (1.25-1.54) |
| Ethnicity | |  |  |  |  |  |  |  |  |  |  |  |  |
|  | Inuit |  | 1382 | 545 | 39.4 (36.3-42.9) | 51.9 (48.2-55.8) |  | 502 | 160 | 31.8 (27.3-37.2) | 59.3 (52.9-66.4) |  | 0.88 (0.76-1.00) |
|  | Non-inuit |  | 184 | 52 | 28.3 (21.6-37.2) | 29.9 (23.0-39.0) |  | 148267 | 105368 | 71.1 (70.6-71.5) | 42.8 (42.4-43.1) |  | 0.70 (0.54-0.91) |
|  |  |  |  |  |  |  |  |  |  |  |  |  |  |
|  |  |  |  |  |  |  |  |  |  |  |  |  |  |
| **Stroke (all)^a^** | |  | 1551 | 2804 | 181 (174-188) | 236 (228-243) |  | 147037 | 404292 | 275 (274-276) | 169 (168-169) |  | 1.40 (1.35-1.44) |
| Sex | |  |  |  |  |  |  |  |  |  |  |  |  |
|  | Males |  | 828 | 1514 | 183 (174-192) | 248 (237-258) |  | 72618 | 204594 | 282 (281-283) | 197 (196-198) |  | 1.26 (1.20-1.31) |
|  | Females |  | 723 | 1290 | 178 (169-188) | 222 (212-233) |  | 74419 | 199698 | 268 (267-270) | 141 (140-142) |  | 1.57 (1.50-1.65) |
| Ethnicity | |  |  |  |  |  |  |  |  |  |  |  |  |
|  | Inuit |  | 1368 | 2562 | 187 (180-195) | 245 (236-253) |  | 498 | 750 | 151 (140-162) | 291 (277-307) |  | 0.84 (0.79-0.89) |
|  | Non-Inuit |  | 183 | 242 | 132 (117-150) | 170 (152-190) |  | 146539 | 403542 | 275 (275-276) | 168 (168-169) |  | 1.01 (0.90-1.13) |
| Males |  | 837 | 48 | 5.73 (4.32-7.61) | 5.60 (4.21-7.46) |  | 73848 | 4307 | 5.83 (5.66-6.01) | 5.70 (5.53-5.87) |  | 0.98 (0.74-1.31) | Males |

**Supplementary table 2 continued**

|  | |  |  | | | |  |  | | | |  |  |
| --- | --- | --- | --- | --- | --- | --- | --- | --- | --- | --- | --- | --- | --- |
|  | |  | Greenlandic residence | | | |  | Danish residence | | | |  | ASIR_GL_/ASIR_DK_ |
|  | |  |  |  |  |  |  |  |  |  |  |  |  |
|  | |  |  |  |  |  |  |  |  |  |  |  |  |
|  | |  | PYRS | Cases | IR (95% CI)  per 100,000 | ASIR (95% CI)  per 100,000 |  | PYRS | Cases | IR (95% CI)  per 100,000 | ASIR (95% CI)  per 100,000 |  |  |
|  | |  |  |  |  |  |  |  |  |  |  |  |  |
|  | |  |  |  |  |  |  |  |  |  |  |  |  |
| **Encephalitis** | |  | 1569 | 106 | 6.76 (5.58-8.17) | 6.75 (5.58-8.16) |  | 149428 | 8265 | 5.53 (5.41-5.65) | 5.28 (5.16-5.40) |  | 1.28 (1.05-1.55) |
| Sex | |  |  |  |  |  |  |  |  |  |  |  |  |
|  | Males |  | 837 | 48 | 5.73 (4.32-7.61) | 5.60 (4.21-7.46) |  | 73848 | 4307 | 5.83 (5.66-6.01) | 5.70 (5.53-5.87) |  | 0.98 (0.74-1.31) |
|  | Females |  | 732 | 58 | 7.93 (6.13-10.3) | 8.06 (6.24-10.4) |  | 75580 | 3958 | 5.24 (5.08-5.40) | 4.87 (4.72-5.03) |  | 1.65 (1.28-2.14) |
|  | |  |  |  |  |  |  |  |  |  |  |  |  |
|  | Inuit |  | 1385 | 95 | 6.86 (5.61-8.39) | 6.92 (5.66-8.45) |  | 503 | 31 | 6.16 (4.34-8.77) | 6.99 (5.03-9.73) |  | 0.99 (0.67-1.46) |
|  | Non-Inuit |  | 184 | 11 | 5.98 (3.31-10.8) | 5.47 (2.95-10.1) |  | 148925 | 8234 | 5.53 (5.41-5.65) | 5.27 (5.16-5.39) |  | 1.04 (0.56-1.92) |
|  |  |  |  |  |  |  |  |  |  |  |  |  |  |
|  |  |  |  |  |  |  |  |  |  |  |  |  |  |
| **Meningitis** | |  | 1565 | 372 | 23.8 (21.5-26.3) | 23.5 (21.2-26.0) |  | 149181 | 20930 | 14.0 (13.8-14.2) | 15.3 (15.1-15.5) |  | 1.53 (1.38-1.70) |
| Sex | |  |  |  |  |  |  |  |  |  |  |  |  |
|  | Males |  | 835 | 208 | 24.9 (21.7-28.5) | 24.8 (21.6-28.4) |  | 73715 | 10990 | 14.9 (14.6-15.2) | 16.6(16.3-16.9) |  | 1.50 (1.30-1.72) |
|  | Females |  | 730 | 164 | 22.5 (19.3-26.2) | 22.0 (18.8-25.6) |  | 75466 | 9940 | 13.2 (12.9-13.4) | 14.1(13.9-14.4) |  | 1.55 (1.33-1.82) |
| Ethnicity | |  |  |  |  |  |  |  |  |  |  |  |  |
|  | Inuit |  | 1381 | 364 | 26.4 (23.8-29.2) | 25.9 (23.3-28.7) |  | 502 | 106 | 21.1 (17.5-25.6) | 21.3 (17.7-25.8) |  | 1.21 (0.98-1.51) |
|  | Non-Inuit |  | 184 | 8 | 4.35 (2.18-8.71) | 5.26 (2.80-9.89) |  | 148680 | 20824 | 14.0 (13.8-14.2) | 15.3 (15.1-15.5) |  | 0.34 (0.18-0.65) |
|  |  |  | 1381 | 364 | 26.4 (23.8-29.2) | 25.9 (23.3-28.7) |  | 502 | 106 | 21.1 (17.5-25.6) | 21.3 (17.7-25.8) |  | 1.21 (0.98-1.51) |
|  |  |  |  |  |  |  |  |  |  |  |  |  |  |
| **Other CNS inf.** | |  | 1569 | 136 | 8.67 (7.33-10.3) | 8.71 (7.36-10.3) |  | 149480 | 9386 | 6.28 (6.15-6.41) | 4.91 (4.80-5.02) |  | 1.77 (1.50-2.10) |
| Sex | |  |  |  |  |  |  |  |  |  |  |  |  |
|  | Males |  | 837 | 63 | 7.53 (5.88-9.63) | 7.60 (5.94-9.71) |  | 73895 | 4521 | 6.12 (5.94-6.30) | 4.95 (4.80-5.12) |  | 1.53 (1.20-1.97) |
|  | Females |  | 731 | 73 | 9.98 (7.93-12.6) | 9.98 (7.94-12.6) |  | 75585 | 4865 | 6.44 (6.26-6.62) | 4.86 (4.71-5.02) |  | 2.05 (1.63-2.59) |
| Ethnicity | |  |  |  |  |  |  |  |  |  |  |  |  |
|  | Inuit |  | 1385 | 125 | 9.03 (7.58-10.8) | 9.36 (7.88-11.1) |  | 503 | 43 | 8.55 (6.34-11.5) | 12.6 (9.86-16.1) |  | 0.74 (0.55-1.00) |
|  | Non-Inuit |  | 184 | 11 | 5.98 (3.31-10.8) | 3.79 (1.81-7.97) |  | 148977 | 9343 | 6.27 (6.15-6.40) | 4.88 (4.77-5.00) |  | 0.78 (0.37-1.63) |
|  |  |  |  |  |  |  |  |  |  |  |  |  |  |
|  |  |  |  |  |  |  |  |  |  |  |  |  |  |

SAH=Subarachnoid hemorrhage, ICH=Intracerebral hemorrhage incl. other nontraumatic intracranial hemorrhages, TIA=Transient ischemic attack, PD=Parkinson’s Disease. Inf.=infections, PYRS=Person-years at risk in thousands, CI=Confidence Intervals, IR=Incidence rates per 100,000 person-years of risk, ASIR=Direct age-standardized incidence rate according to WHO standard population, ASIR_GL_/ASIR_DK_= Ratios between Greenlandic and Danish ASIRs, GL=Greenland, DK=Denmark.

^a^In stroke (all) only the first incident diagnosis of either SAH, ICH, TIA or ischemic stroke is included.
